# Identification of Soybean MicroRNAs Involved in Soybean Cyst Nematode Infection by Deep Sequencing

**DOI:** 10.1371/journal.pone.0039650

**Published:** 2012-06-27

**Authors:** Xiaoyan Li, Xue Wang, Shaopeng Zhang, Dawei Liu, Yuxi Duan, Wei Dong

**Affiliations:** 1 Beijing Institute of Genomics, Chinese Academy of Sciences, Beijing, China; 2 Graduate University of Chinese Academy of Sciences, Beijing, China; 3 Nematology Institute of Northern China, Shenyang Agricultural University, Shenyang, China; 4 Beijing Genomics Institute, Hangzhou, China; East Carolina University, United States of America

## Abstract

Soybean cyst nematode (SCN), *Heterodera glycines*, is the most devastating pathogen of soybean worldwide. MicroRNAs (miRNAs) are a class of small, non-coding RNAs that are known to play important role in plant stress response. However, there are few reports profiling the miRNA expression patterns during pathogen stress. We sequenced four small RNA libraries from two soybean cultivar (Hairbin xiaoheidou, SCN race 3 resistant, Liaodou 10, SCN race 3 susceptible) that grown under un-inoculated and SCN-inoculated soil. Small RNAs were mapped to soybean genome sequence, 364 known soybean miRNA genes were identified in total. In addition, 21 potential miRNA candidates were identified. Comparative analysis of miRNA profiling indicated 101 miRNAs belong to 40 families were SCN-responsive. We also found 20 miRNAs with different express pattern even between two cultivars of the same species. These findings suggest that miRNA paly important role in soybean response to SCN and have important implications for further identification of miRNAs under pathogen stress.

## Introduction

The soybean cyst nematode (SCN), *Heterodera glycines* Ichinohe, is an obligate sedentary endoparasite that causes extensive damage to soybean, *Glycine max (L.) Merr*., worldwide and accounts for over one billion dollars loss annually in the US [1]. Current nematode control strategies include nematicides, crop rotation and resistant cultivars, but each has serious limitations. The lack of understanding of nematode virulence has hampered the ability to devise novel management tactics [2]. Advances in genomics during the last years provide opportunities for us to investigate molecular mechanism of the *G. max-H. glycines* system. Nematode genome sequencing projects and comparative analysis contribute to insight the nature of the evolution of plant parasitism [3], [4], [5]. Laser capture microdissection (LCM), a refined approach to obtain homogeneous cell samples, are enabling the discovery of the genes and pathways contributing to the formation of unique, highly specialized feeding cells induced within plant roots by parasitic nematodes [6], [7]. Emerging tools, such as microarray, RNA-seq, RNA interference hold tremendous potential to uncover the mechanism of soybean-SCN interactions [8], [9], [10], [11], [12]. Previous studies indicate that plants reprogram gene expression at the transcriptional, post-transcriptional and post-translational levels to reduce the damage to stress. Increasing evidences unraveling that host endogenous smallRNAs are essential in this gene expression reprogramming process [13].

MicroRNAs (miRNAs) are a class of 21–24 nucleotide long non-coding RNAs that negatively regulate gene expression in animals and plants [14]. MiRNAsrole in plant was first demonstrated in plant growth, development, hormone signaling, and RNA metabolism [15], [16]. Recently, it was reported that miR393 was induced by a bacterial flagellin-derived PAMP, Flg22 in *Arabidopsis*. MiR393negatively regulates the expression level of the F-box auxin receptor genes and restricts *Pseudomonas syringae* growth. Combined with race-specific resistance test, the results suggest that miR393 has a role in imparting basal resistance but not race-specific resistance [17]. Researchers find 10 miRNAs are down-regualted in loblolly pine response to rust fungus [18]. Two miRNAs, bra-miR158 and bra-miR1885, were greatly upregulated when *Brassica rapa* was infected by Turnip mosaic virus [19]. Genome-wide small RNA profiling analysis from nodules or from roots inoculated with symbiotic rhizobia identified a number of nodulation-associated miRNAs from different legumes [20], [21], [22], [23], [24]. Mtr-miR166, Mtr-miR169 of *M. truncatula* and three soybean miRNAs, Gma-miR482, Gma-miR1511, Gma-miR1512 have been functionally analysed and associated to rhizobial symbiosis [25], [26], [27]. These increasing evidences show that miRNAs are involved in plants response to biotic stresses.

To date, many conserved or novel miRNAs have been identified in soybean by computational analysis or high-throughput sequencing [28], [29], [30], [31], [32], [33]. MiRNAs related to *Bradyrhizobium japonicum*, *Phytophthora sojae* response are obtained by comparative analyzed [20], [34]. However, there is no report of miRNAs associated with SCN infection. Here, using solexa analyzer, we identified a diverse set of small RNAs which response to SCN infection in both resistance and susceptibility soybean roots. Totally, 21 new miRNAs belong to 19 families were obtained. Moreover, we also found that 40 soybean miRNA families showed differential expression level in response to soybean cyst nematode infection. Some potential targets of these miRNAs were stress responsive genes.

## Methods

### Plant material and RNA isolation

Glycine max, cultivar “Harbin xiaoheidou” (HB) were chosen as a soybean cyst nematode race 3 resistant standard and “Liaodou 10” (L10) were used as SCN race 3 sensitive standard. The plants were grown in greenhouse, cyst inoculation samples were propagated on SCN race 3 infected-soil which was collected from the pilot field of Shenyang Agriculture university, control samples were grown on autoclaved blank soil. Root samples were collected 30 days after seedlings emerge. All the tissues were harvested and immediately frozen in liquid nitrogen and stored at −80°C. 80 mg of plant tissues were ground to powder with liquid Nitrogen. Total RNA was isolated from soybean root samples with TRIzol reagent (Invitrogen, USA) according to the manufacturer's protocol. Total RNA was treated with DNaseI (NEB, USA) for 30 min at 37°C, and then purified by ethanol precipitation. The RNA samples were qualified by Agilent 2100.

### Small RNA libraries construction and SBS sequencing

Small RNAs isolation and library construction were performed as described by Hafner Markus, *et al* [35]. Firstly, total RNA was size fractioned on 15% TBE urea polyacrylamide gel and 18–30 nt fraction was collected. The 5′ RNA adapter was ligated to the RNA pool with T4 RNA ligase. Ligation products were gel-purified. Then ligated with 3′RNA adapter and purified. Small RNAs with both side adaptors were subjected to reverse transcription, 15 cycles of PCR reaction was performed to produce sequencing libraries, and the products were gel-purified.

Small RNAs samples were sequenced using Illumina Genome Analyze II at Beijing genomics Institute (Shenzhen). 10 pM of each sample was used for cluster generation. After hybridization of sequencing primer, 35 cycles of base incorporation were carried out on the 1G analyzer. More than 16 million 35 nt reads were generated for each sample.

### Data analysis and new miRNAs identification

Image analysis and base calling were performed using Illumina Pipeline. Low quality reads (tags with unknown nucleotide ‘N’), empty tags (sequence with only adaptor sequence), tags less than 17 nt, and low complexity tags were trimmed with our own perl script. Removed 3′ and 5′ adapter, purified tags were mapped to the soybean genome sequence (http://www.phytozome.net/cgi-bin/gbrowse/soybean) using SOAP v1.11 (BGI). Tags that perfectly matched soybean genome were obtained for subsequent analysis. These tags were used to search for Rfam database (http://rfam.sanger.ac.uk/), unique reads mapped to tRNA (transfer RNA), rRNA (ribosomal RNA), snRNA(small nuclear RNA), snoRNAs (small nucleolar RNAs), known miRNAs (microRNAs) were removed. Repeat overlapping sequences were annotated as repeat-associated small RNAs. Tags matched to gene exon region were excluded from further analysis.

Uni-tags resulted from the pre-processing steps, with copy number more than one and less than 24 nt were tested by Mireap developed by BGI (Shen Zhen) to predict novel miRNAs. The algorithm includes two dependent parts. First of all, candidate miRNA sites are screened out from breakpoints defined by small RNAs mapping. At each genomic locus, two sequences covering the read were extracted for secondary structure analysis, one sequence extending 160 nt upstream and 20 nt down-stream from the read, and the other covering 20 nt upstream and 160 nt downstream of the read. Then, a minimal stringent criterion was used to select miRNA candidates. The secondary structure must have a hairpin with at least 18 paired nucleotides in its stem region. The free energy of the hairpin should be less than -18 kCal/mol. MiRNA and miRNA* had to reside in different arms of a hairpin structure, each with no more than 6 unpaired bases. The maximum bulge over the miRNA/miRNA* duplex was not more than 4 bases, and asymmetry of the miRNA/miRNA* duplex equal to or less than 4. In addition, sequencing of both miRNA and miRNA* required that the miRNA/miRNA* duplex had 3′ overhangs at both ends, a typical feature of Drosha and Dicer processing [36]. The resulting set of sequences and their respective RNA structures were further analyzed to distinguish genuine miRNA precursors from other RNAs containing similar RNA structures.

Mature miRNA sequences were used as queries to search for potential target mRNAs in the *Glycine max* database (http://compbio.dfci.harvard.edu/index.html, DFCI gene index release 16) using the web-based computer psRNATarget Server (http://biocomp5.noble.org/psRNATarget/). Results from these analyses were inspected on the Phytozome to obtain the loci and protein annotation. The rules used for miRNAs target gene prediction were based on suggestions by Allen *et al*. and Schwab *et al*. respectively [37], [38]. No more than 4 mismatches between the sRNA and target were allowed. The minimum free energy (MFE) of the miRNA/target duplex should be > = 74% of the MFE which the miRNA bound to its perfect complement [39], [40].

### Differential expression analysis of miRNAs

The frequency of miRNA was normalized to TPM (number of transcripts per million clean tags) in order to compensate for variable numbers of tags generated for each sample. The fold-change between SCN-infected and control sample were calculated. To avoid divided by 0, we use 1 instead of tag that was not detected in sample. Then the statistically analysis was performed according to Poisson distribution.

(1) Normalization criterion:

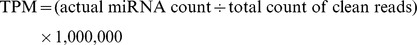

(2) Fold-change criterion:

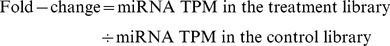

(3) The P-value calculated formula:

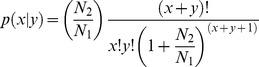


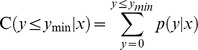


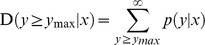



### Quantitative PCR analysis

The profiles of 16 different expressed miRNAs were assayed by Real-time PCR. About 200ng qualified total RNA for each sample were used for this analysis.

The miRNA gene specific reverse transcript stem-loop primers were designed using Primer 5.0 following the method described by Varkonyi-Gasic *et al* [41]. The reverse transcript reaction was performed with Superscript II (Invitrogen, USA) following the the manufacturer's protocol. Reverse transcript products were used as template for Real-time PCR and all reaction were assayed in triplicates. The reactions were performed in Stepone RT-PCR machine (Applied Biosystems, USA) using SYBR Premix ExTaq kit (Takara, China). PCR cycling began with template denaturation and hot start Taq activation at 95°C for 1 min, then 40 cycles of 95°C for 5 sec, and 60°C for 30 sec performed and data collected during each cycle at the 60°C extension step. The U6 snRNA was selected as a reference gene for normalization. Relative quantitation of the miRNAs amplified was performed by the comparative ΔΔC_T_ method. The threshold cycle (C_T_) indicated the fractional cycle number at which the amount of amplified target reaches a fixed threshold.
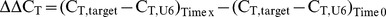



Time x represents any time point and Time 0 represents the expression of the target gene normalized to U6 [42]. The amount of relative gene expression can be obtained.

## Results

### High-throughput sequencing of soybean small RNAs

To investigate the role of soybean miRNAs in response to soybean cyst nematode (SCN) infection, two soybean cultivars, Liaodou10 (L10), a soybean cyst nematode race 3 susceptible cultivar and Harbin xiaoheidou (HB) which resistant to SCN race 3 were selected for this study. Totally, four small RNA (sRNA) libraries were constructed from the two cultivars. The SCN-infected sRNA libraries from HB and L10 roots were named SHB, SL10 respectively. While sRNAs libraries from un-infected roots of HB and L10 were constructed as control, which designated as SHBC and SL10C. These four soybean root small RNA libraries were sequenced by Illumina Genome Analyze II, yielding a total of 69,861,808 sRNA raw reads. After removing the low quality reads, 11,831,532, 10,980,930, 10,494,778 and 11,238,552 clean reads were obtained for SHB, SHBC, SL10 and SL10C, respectively. These sRNAs were consisted by 11,317,750 unique sequences as shown in table 1, and these reads were searched against the public soybean genomic and expressed sequence tags using SOAP program, leading to 5,103,481 genome-matched reads [43].

**Table 1:** Statistics of sequenced tags.

sRNAs	SHB	SHBC	SL10	SL10C
Total raw reads	18287677	18472464	16514309	16514309
clean reads	11831532	10980930	10494978	11238552
Unique reads	2469340	2950719	3577791	2319900
sRNAs mapping to genome	8865700	8013950	7337893	5977971
unique reads mapping to genome	1237317	1613828	977395	1274941

Majority of sRNAs were sequenced only a few times although some sRNAs were highly abundant and present thousands of times in one sample. About 36% of the sRNAs were sequenced only once suggests soybean roots have large and diverse sRNAs population.

**Figure 1:**
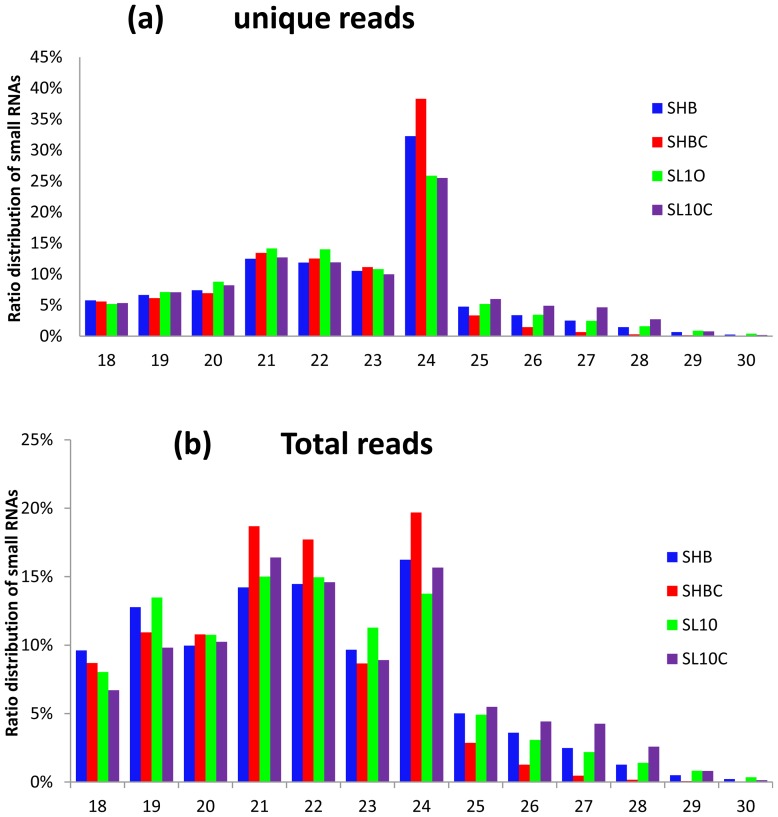
Length distribution of soybean roots sRNAs. (a) Ratio of unique sRNAs reads vs size. (b) Ratio of total sRNAs reads vs size.

More than 90% of clean reads in this study were longer than 18 nt. Size distributions based both on total abundances and unique reads were assessed and shown in figure 1. For total abundance, more than 75% sRNAs were 19–24 nt in length, with 21, 22, 24 nt being the major length classes. For the proportion of unique reads, the 24 nt reads were prevailing in all four libraries and the 21, 22 nt reads were less abundant. Analysis revealed that there is a variation in abundance of sRNAs, the 21, 22 nt sRNAs showed higher redundancies than 24 nt sRNAs. The observation indicated the differently expression pattern of distinct categories of sRNAs in the same sample. This result was consistent with those of *Citrus rifoliate*, *Arabidopsis*, *Medicago truncatula* [44], [45], [46].

The common/specific sRNAs in SHB vs SHBC and SL10 vs SL10C were analyzed for total and unique tags, the results highlight the fact that sRNAs common in different libraries had more abundance than those library specific sRNAs (Table S1).

### Identification of known miRNAs

In this study, miRBase release 18.0, which contains 391 soybean mature miRNAs, was searched for soybean known miRNAs [47]. A total of 364 known soybean mature miRNAs were identified in at least one library, among which 283 were sequenced in all 4 four libraries and 53 miRNAs were detected only in one or two libraries, which was summarized in table 2. There were 27 known soybean miRNAs, containing 7 conserved miRNAs and 20 fabaceae or soybean specific miRNAs, were absent in our data set (Table S2). This is probably due to low expression levels or stage-/tissue- specific RNA transcription. Two of reported miRNA*, gma-miR1507c* and gma-miR1516* were also identified.

**Table 2:** Summary of small RNAs mapping to known miRNAS.

	miRNAs	miRNA*	hairpin	unique_sRNAs_match_hairpin	total_sRNAs_match_hairpin
known*	391	4	362	-	-
SHB	323	1	298	3776	740041
SHBC	328	1	303	4352	1317136
SL10	311	2	287	2831	454032
SL10C	335	1	309	3375	818645

* : Soybean miRNAs registered in miRBase.

Gma-miR1507ab was the most abundant miRNA in all the 4 samples in our study, followed by members of MIR156. The top 30 abundant miRNAs which occupied more than 85% of expressed miRNA tags were generally unanimous between samples. Most of the top 30 abundant miRNAs were conserved miRNAs such as, members of MIR156, MIR166 and MIR168, while three legume specific miRNAs, miR1507, miR1509 and miR1510 were highly abundant in all four libraries. Further analysis revealed that conserved miRNAs had relative high abundant in general, this observation suggested that conserved miRNAs may be essential for controlling basic cellular and developmental in plants. More than half of the miRNAs showed very low levels of expression, with fewer than 100 reads.

Within the tags that mapped to known miRNAs over 55% have length 21 nt, followed by 20 nt and 22 nt reads. Among the miRNA reads, 24 nt length was less than 1%. This result was consistent with current understanding. The canonical miRNAs are 21 nt length, while canonical siRNAs are 24 nt length [48]. About 94% of these miRNA reads start with a uracil contrast to 30% of all small RNAs. These distribution were examined in all four samples individually, there were no obvious difference among them.

We observed there are diverse isoforms of mature miRNAs from the same precursor and the 3′ ends of miRNA showed stronger heterogeneity than the 5′ ends. As exemplified by miR1509a in Figure S1, the highlighted 3′ ends isoform were highly accumulated. Some most abundance tags mapped to hairpin were not previous annotated miRNAs. Gma-miR156b is an extreme example, the former annotated miRNA were not detected in all 4 samples, indicating the tagt0006055 should be regarded as the final functional molecular.

### Prediction and validation of novel soybean miRNAs

Small RNAs perfectly matched to soybean genome sequences were searched against Rfam, ncRNA (non-coding RNA) of NCBI, EST database to eliminate other ncRNAs, such as rRNA, tRNA, snRNA, snoRNA and degradation products of protein-coding transcripts. Small RNAs were aligned to repeat associated RNA to find matched tags in the data set. With the analysis, 3,571,130 tags named unann were obtained for novel miRNA detection (Table S3). Characteristic hairpin structures of those reads were explored using Mireap. Then precursors were manually checked according the criteria described by Blake C. Meyers, *et al* [36]. Twenty two new soybean miRNAs not previously reported were identified by the analysis, named temporarily in the form of soy_number in table 3. Ten of the 22 new miRNAs had length 21 nt, followed by 6 miRNAs with 22 nt length. The minimum free energy (MFE) for hairpin structure of miRNA precursor were lower than -25 kcal/mol. Stem-loop structures of miRNAs precursors were predicted by MFOLD.

**Table 3:** Novel miRNA candidates predicted from miRNA precursor.

miRNA_id	Sequence	Length	Location	Number of loci in the soybean genome	SHB	SHBC	SL10	SL10C
soy_1	TCGGACCAGGCTTCATTCCCT	21	intergenic	1	1614	2522	797	1444
soy_2	CCTCGGACCAGGCTTCATTCC	21	intergenic	1	6	10	8	37
soy_3	TCGGACCAGGCTTCATTCCCTT	22	intergenic	1	6	16	2	6
soy_4	TGAAGTTCGTAGATGGAATCA	21	intergenic	1	10	19	0	0
soy_5	AGATATGATGATGTTTATCCA	21	intergenic	1	90	97	35	37
soy_6	ATGCGACTTTTGGAGGACAGC	21	intergenic	1	0	11	0	0
soy_7	CAGGAGCTGGTGGGACATTCCT	22	CDS	1	0	5	6	0
soy_8	CAGAAATTGTAAGCGAGGGAG	21	intergenic	1	0	8	0	6
soy_9	AGGGACAGGGCAGATAAGTT	10	intergenic	3	21	0	0	0
soy_10	TCTGATCAGTTTGAGGCTAAGC	22	intergenic	7	12	0	0	0
soy_11	AGGAGAGTTGCTAGCACTTGT	21	intergenic	1	15	0	48	5
soy_12	TTTTGTGTCGTGAAGCTTTTG	21	intergenic	2	48	0	11	8
soy_13	AACTGTTGCAATGTAGAGTAGAA	23	CDS	1	14	0	0	0
soy_14	GATCGGAACCTCTTCTGCGGCG	22	CDS	1	0	0	20	0
soy_15	AGCTTGGACTAGGGGGCACATCT	23	intergenic	1	0	0	14	0
soy_16	TCACAATGAGGATACAAAAGCT	22	intergenic	1	0	0	79	0
soy_17	GAGCAATCGTGAAGGAGACA	20	intergenic	1	0	0	11	0
soy_18	ACCCGAATCCGGTGCCAATCCC	22	CDS	1	0	0	11	0
soy_19	AACTGGAGGGAGGAATTTCT	20	intergenic	3	6	0	0	9
soy_20	TCTGATGGGCTAGAGATATG	20	intergenic	1	0	0	0	11
soy_21	AGCAGAACGTCATTCTCTTGG	21	intergenic	1	0	0	10	16

Novel miRNA candidates were further assigned to miRNA families using sequence similarity to other known miRNA in miRBase database. Among the 21 novel miRNAs, 18 miRNAs have not been found in eukaryotes, except soy_1, soy_2, soy_3. All the three miRNAs were members of MIR166, which conserved in both monocots and dicots. The 18 miRNAs without homolog in database, along with the known soybean miRNAs were used for multiple alignments using ClustalW and the miRNA families were assigned based on the dendogram tree [49]. None of the miRNAs was assigned to families based on sequence similarity, while the miRNAs will get families with further study.

**Figure 2:**
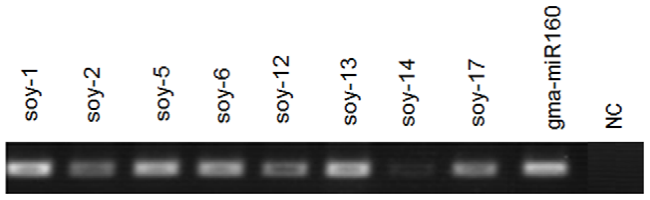
Step-loop RT-PCR for novel miRNAs. Gma-miR160: the positive control, NC: Water was added instead of RNA in the reverse transcript reaction, the negative control.

To validate the predicted new miRNAs, stem-loop RT-PCR were performed to examine whether the miRNAs were expressed in the soybean roots. Eight miRNAs which had more abundance were selected to confirm the analysis. Primers used in this experiment are showed in S5. The PCR products were about 60bp in length, and all the 8 miRNAs were found to be expressed in the soybean roots (Figure 2).

### SCN-infection associated miRNAs

**Figure 3:**
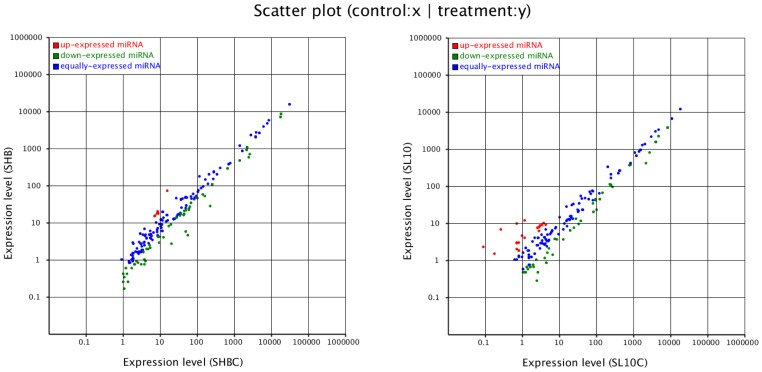
Scatter plot of miRNA expression level. Expression levels are normalized to TPM. Data points lower- or upper- the slope line represent down- or up- regulated miRNAs in panel. The changes in up- and down-regulated miRNAs are greater than 2 fold.

Although the absolute expression level of miRNA is useful, identification of differential expression profile at the whole genome level in response to endogenous cues or stress is often desirable to detect miRNA function in particular cell processes. The sequencing frequencies for miRNAs in the four libraries were used as an index for estimating the relative abundance, expression level between SCN-infected soybean root and un-infected root were compared based on the “transcripts per million”(TPM) of miRNAs (Figure 3).

**Figure 4:**
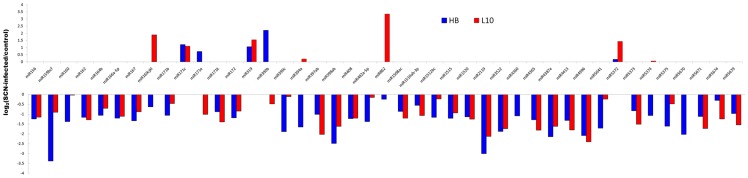
Differential expressed miRNAs in response to SCN. miRNA with expression changes greater than 2 fold and P-value lower than 0.01 at least in one cultirvar.

We used fold-change values ≥ 2 or ≤0.5 combined with P-value <0.01 as the threshold, a total of 101 miRNAs belong to 40 families were identified to be significantly differentially expressed in at least one cultivar, the results were showed in Figure 4. Most of the differentially expressed miRNAs were down regulated during the SCN-infection and only 6 miRNAs were up-regulated. Thus, down-regulation of miRNAs appeared to be important in SCN infection. MiR171c and miR319 were highly induced by SCN infection in both cultivars, expression of miR390b was induced in HB specific while miR862, miR5372 and four members of miR169 were SCN-infection induced only in L10. MiR159b, miR398 and miR2119 showed the highest alternation with 12, 8.5 and 8 fold changes in HB while miR862b increased to 25 fold after SCN infection was the highest changed miRNA in L10. There were 16 miRNA families which were co-regulated in both two genotypes, among them, miR156, miR162, miR166a, miR167, miR319, miR397, miR398, miR408 were conserved miRNAs between plants species, two miRNA families, miR2119 and miR3522 were conserved in fabaceae, while miR1520, miR4365, miR4387, miR4413, miR4996 and miR5671 were soybean specific miRNAs. The results indicated that both conserved miRNAs and soybean specific miRNAs participated in nematode defense. HB, the SCN race 3 resistant cultivar, specific response miRNAs contained 15 members, while there were 9 susceptible cultivar L10 specific response miRNAs.

Notably, some members of one miRNA family had varied frequencies and the members showed differential expressed pattern during SCN-infection. MiR1507 had three members, gma-miR1507ab frequency was more than 40 times of gma-miR1507c. MiR1507ab was down-regulated after SCN-infection while miR1507c was induced by nematode infection. We also found that members of MIR390 and MIR171 had different expression pattern showed in Table S4. Differential expressed pattern indicating the function divergence in miRNA families.

**Figure 5:**
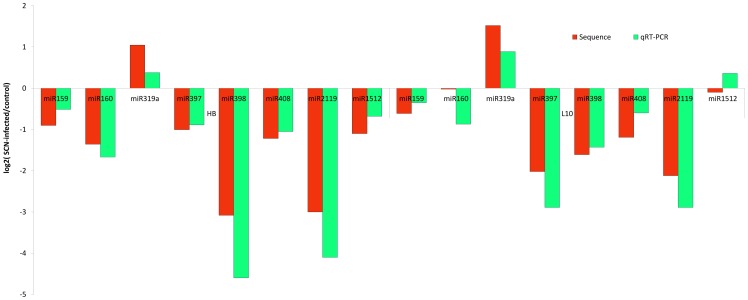
qRT-PCR results. qRT-PCR confirming express pattern of miRNAs.

To confirm the Solexa sequencing results, we performed qRT-PCR to further validate several miRNAs that were detected as differential expressed. We found that most of the qRT-PCR results were consistent with the deep sequencing data (figure 5). However, there were differences between the sequencing results and qRT-pCR data, the log_2_ ratio of gma-miR1512 in L10 was −0.1 with sequencing data, but the qRT-PCR ratio was 0.36, this could been due to sequence bias induced by the small RNA library construction or to the different normalization methods in this two strategy.

### Targets prediction of soybean miRNAs

MiRNAs regulate expression of specific gene via hybridization to mRNA transcripts to promote RNA degradation, inhibit translation or both [50]. We predicted the potential targets of miRNAs using the webserver, psRNATarget [51]. For the miRBase registered soybean miRNAs, a total of 293 target genes were identified showed in Table S5. Combined with previous studies, we investigated that most target of conserved miRNAs were transcript factors which involved in plant growth and stress responses. Some of miRNA families, such as MIR156, MIR160, MIR164, MIR393, MIR396, MIR1510 were conserved in their targets. The targets prediction results of MIR1507, MIR390 and MIR171 were checked manually, gma-1507a and gma-1507b had the same target genes, while potential targets of gma-1507c were totally different. This was consistent with the expression pattern between miRNAs. Members of MIR390 and MIR171 come to the same results.

Among the 21 novel identified miRNAs, 16 miRNAs belonged to 14 families had predicted targets, the results were showed in table 4. Soy_5, soy_8, soy_13, soy_17, soy_20 target only one locus, while other miRNA candidates had multiple distinct targets genes. Soy_1, soy_2 and soy_3 were members of MIR166, targets of the three miRNA candidates were HD-ZIP transcript factor which were conserved with other miRNA members. Function of new miRNA tragets were diverse and were not enriched in transcript factors compared to conserved miRNAs. Calmodulin, serves as an intracellular Ca^2+^ receptor and mediates the Ca^2+^ regulation of cyclic nucleotide and glycogen metabolism, secretion, motility and Ca^2+^ transport, also a dynamic component of the mitotic apparatus. Calmodulin was detected as soy_9 potential targets indicating the miRNA may participate in cellular regulation [52].

**Table 4:** Predicted targets of novel miRNAs.

miRNA	Target Gene	UPE	Inhibition	Target Gene Description
soy_1,2,3	Glyma07g01940	23.681	Cleavage	HD-Zip protein
	Glyma09g02750	19.231	Cleavage	HD-Zip protein
	Glyma05g30000	19.236	Cleavage	HD-Zip protein
	Glyma02g39210	17.713	Translation	zinc finger protein
soy_4	Glyma10g32900	12.649	Cleavage	H1-2flk
	Glyma07g15820	22.974	Cleavage	MYB transcription factor
	Glyma18g39740	22.67	Cleavage	MYB transcription factor
soy_5	Glyma16g05350	15.011	Cleavage	GA protein
soy_6	Glyma15g39160	15.377	Cleavage	Cytochrome P450 monooxygenase
	Glyma07g34530	10.633	Cleavage	HSP20-like chaperone
soy_8	Glyma14g36930	8.55	Translation	CONSTANS-LIKE
soy_9	Glyma13g03910	9.724	Cleavage	Calmodulin
	Glyma14g04460	14.466	Cleavage	Calmodulin
	Glyma06g45170	13.597	Cleavage	Calmodulin
	Glyma02g44350	18.69	Cleavage	Calmodulin
soy_11	Glyma19g00710	22.652	Cleavage	lipid-transfer protein
	Glyma02g40720	19.741	Cleavage	Phytoene synthase
soy_12	Glyma10g44170	12.37	Cleavage	AGO
	Glyma13g39500	17.158	Cleavage	AGO
	Glyma20g06210	19.293	Translation	NAC
	Glyma13g26820	14.096	Cleavage	NAC
soy_13	Glyma17g10450	10.291	Cleavage	Nitrate transporter
soy_16	Glyma10g43810	10.252	Cleavage	hPP2c
	Glyma18g02430	22.655	Cleavage	TGA-type basic leucine zipper protein
	Glyma01g09530	14.303	Translation	NADH dehydrogenase subunit 4L
soy_17	Glyma12g02460	13.017	Translation	Ubiquitin carrier protein
soy_19	Glyma03g24170	20.866	Cleavage	TcC31.32
	Glyma09g01730	12.902	Cleavage	2OG-Fe(II) oxygenase
	Glyma13g39790	19.776	Translation	ATP-binding cassette sub-family member
soy_20	Glyma13g42330	18.656	Cleavage	Lipoxygenase-4
soy_21	Glyma14g03650	20.748	Cleavage	Auxin response factor
	Glyma19g38720	19.934	Cleavage	Digalactosyldiacylglycerol synthase

## Discussion

High-throughput sequencing is an efficient strategy to study miRNAs at the whole genome level and has dramatically expanded the number of miRNA families known to exit in plants. As to soybean, 391 miRNAs have been well annotated by deep sequencing and registered in miRBase database [47]. In present study, we sequenced small RNA libraries of two genotype soybean root treated by soybean cyst nematode, totally got 69,788,759 reads and 11,317,750 unique reads. The large amount of small RNA data revealed a diverse and complex small RNA population. Moreover, the large data set allows us to identify miRNAs with low abundance. In total, 364 known miRNAs and 21 novel miRNAs were detected, 171 of them had frequency less than 20. Combined with the previous report, 40% the soybean miRNAs are belonged to soybean or lineage specific, while roughly 70% known *Arabidopsis* miRNA families lack apparent homologs in outside of the brassicasseae [53]. This result indicates that miRNAs discovered in soybean has not reached saturation. Novel miRNA detected in this study showed relatively low expression levels, which was consistent with previous studies [54], [55], [56]. Thus much larger dataset are needed to identify new miRNAs than previous studies.

In our study, gma-miR1507ab was the most abundant miRNAs in all four samples followed by miR1509, miR1510 and miR2118. Although miR1509a and miR2019 were annotated other than 22 nt, based our data, previous register should be corrected. In *M. truncatula*, peanuts and common beans, miR1507, miR1509, and miR2118 were highly abundant 22-nt miRNAs [57]. The conservation and abundant expression of these 22 nt in legumes indicated unique evolution ways of these lineage-specific miRNAs. More interestingly, we found that all the four miRNA families were regulators of legume NB-LRR-coding genes. Furthermore, potential targets of miR5039, miR5041, miR5374, miR5376, miR5668 were plant NB-LRR transcripts. Until now, the only function of plant NB-LRR proteins is recognize pathogen effectors and activate defense responses [58]. The results implicating that the specialized group of miRNAs in legume target NB-LRR genes are crucial regulator to some legume specific plant defense to pathogens.

MiR319 was shown to be drought-responsive in *Arabidopsis*, rice, and sugarcane, while we firstly report miR319 was involved in biotic stress [61], [62], [63]. Gma-miR319 was the single up-regulated conserved miRNA family in both two cultivars. In contrast to deeply conserved miRNA families, most young miRNAs were weakly expressed, more divergent, and tend to lack targets. Thus, function information about non-conserved miRNA is rare. The 20 legume- or soybean specific miRNAs related to SCN infection were identified in current study, suggesting a number of non-conserved miRNAs function as regulators of genes in plant genomes, and play an important role in plant pathogen defense. The complex plant response to SCN infection spans the early migratory stages during penetration and migration through roots into the later sedentary stages of syncytium induction and feeding which have inspired numberous studies to identify genes involved in SCN. Here we report the miRNAs related to SCN infection. In previous study, 43 SCN-responsive miRNAs were identified. More than 80% of miRNAs were suppressed by SCN infection in both cultivars. Similarity, 10 of 11 miRNAs were down-regulated in the stem galls induced by the rust fungus *Cronartium quercuum* f. sp. *Fusiforme* [18]. While in *Arabidopsis*-*H. schachtii* interaction study, all the 14 differentially expressed miRNAs were statistically significantly down-regulated at the 4 dpi (day post-inoculation) while at 7 dpi, 5 miRNAs were up-regulated and 7 were down-regulated [59]. Indicating the tested miRNAs have complex expression profiles at different stage of cyst nematode infection which is consistent with gene expression pattern during SCN-infection [9], [10]. Targets of SCN-responsive miRNAs include transcription factors, general stress-responsive genes, disease resistance responsive proteins, PR proteins. All these genes expression pattern were checked in previous studies, about 50% miRNAs showed negative correlations between miRNA accumulation and target gene mRNA abundance [60]. These discrepancies suggest that other regulatory mechanisms could be involved in regulating these genes.

MiR319 was the single up-regulated conserved miRNA family in both two cultivars, which was shown to be drought-responsive in *Arabidopsis*, rice, and sugarcane [61], [62], [63]. In tomato, miR319 was involved in *Solanum lycopersicum*, and showed opposite expression patterns in leaves [64]. The diverse functions of miR319 were due to the target gene TCP that presumably control cell divisions in stress response. Interestingly, during the same stress, several miRNAs had differential express pattern in the two cultivars. MiR169 was up-regulated in the sensitive cultivar, but down-regulated in the tolerant. MiR169 targeted nuclear factor Y subunit, which can regulate expression level of some stress-responsive genes [65]. MiR169 guided regulation of these transcription factors appeared to be important in variety of abiotic stress, including drought, cold, salinity, UV-B radiation, and heat stress [61], [62], [63], [65], [66]. Furthermore, *MtHAP2-1*, a new transcription factor of the CCAAT-binding family identified in *M. truncatula*, is regulated by miR169. *MtHAP2-1* is essential for nodule meristematic persistence in *M. truncatula*, and that miR169 confers spatial and temporal accuracy of nodules development [67]. Contrarily, gma-miR390b was induced by SCN in HB and suppressed in L10. MiR390 was involved in auxin signaling and regulated root growth [68]. Gma-miR390c were down-regulated in HB but without significant changes in L10. Expression patterns of MIR390 members are different even in single cultivar which indicating function diversity of this miRNA family. With other 15 miRNAs up- or down-regulated only in one cultivar, the genotype-specific regulation of miRNAs might be part of the reason why the two soybean cultivars had differential tolerance to soybean cyst nematode.

In summary, our study have identified miRNAs form soybean roots, analyzed their expression pattern during soybean cyst nematode infection and predicted the putative targets of these miRNAs. Further function characterization of SCN-responsive miRNAs and targets will contribute substantial new knowledge to our understanding of the complex soybean-SCN pathosystem.

## Supporting Information

Figure S1 **Diversification of mature miRNA from precursors.** Detected diverse isoforms of soybean miRNA, the most abundant sRNA are underline in red, sRNAs in red frame are annotated mature miRNA in miRBase.
(TIF)

Table S1 **Statistics of common sRNAs from control and SCN-infected.** Statistics of common sRNAs from control and SCN-infected sample in both total and unique reads.
(XLSX)

Table S2 **Known miRNAs identified in** ***Glycine max.*** Soybean known miRNAs identified frequency in each sample.
(XLSX)

Table S3 **Annotation results of sRNAs.** Reads abundance of various classification of small RNAs in each sample.
(XLSX)

Table S4 **Express pattern diverse in miRNA families.** Different members of miRNA family with differential expressed pattern during soybean response to SCN.
(XLSX)

Table S5 **Predicted targets of soybean miRNAs.** Potential targets of miRNAs predicted using psRNATarget.
(XLSX)
