# Supplementary figures and images for: Identification of Soybean MicroRNAs Involved in Soybean Cyst Nematode Infection by Deep Sequencing
Source: PLoS One. 2012 Jun 27;7(6):e39650. doi: 10.1371/journal.pone.0039650 (PMC3384596; doi:10.1371/journal.pone.0039650)

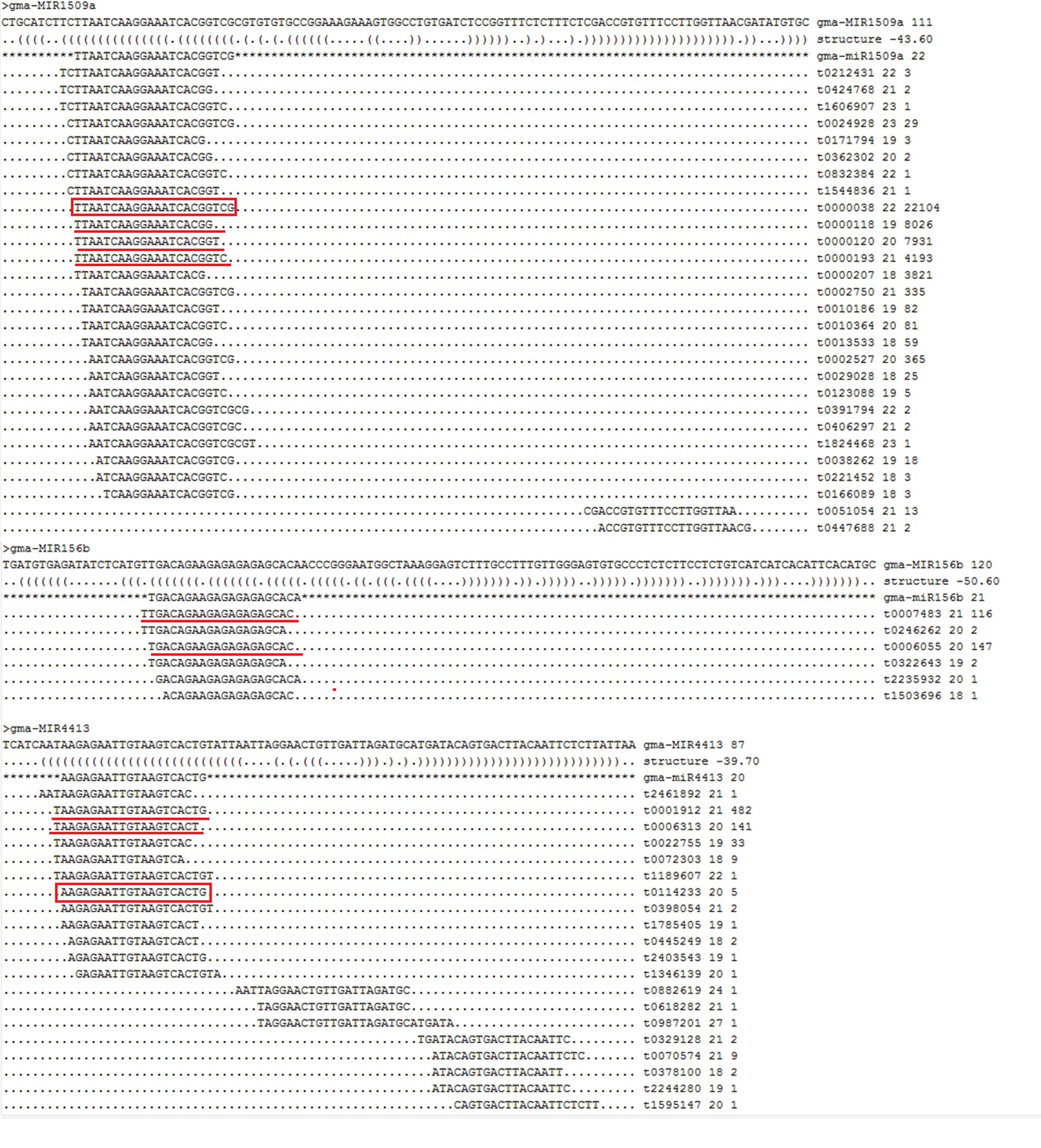

Supplement: Figure S1 — Diversification of mature miRNA from precursors. Detected diverse isoforms of soybean miRNA, the most abundant sRNA are underline in red, sRNAs in red frame are annotated mature miRNA in miRBase. (TIF) [file pone.0039650.s001.tif]
